# The possibilities and limits of insurance as governance in insuring pandemics

**DOI:** 10.1057/s41288-023-00291-z

**Published:** 2023-03-21

**Authors:** Qihao He, Michael Faure, Chengwei Liu

**Affiliations:** 1 College of Comparative Law, China University of Political Science and Law, Beijing, China; 2 Maastricht University, Maastricht, The Netherlands; 3 Erasmus University Rotterdam, Rotterdam, The Netherlands

**Keywords:** Pandemic risks, Insurability, Insurance as governance, Limits of insurance, Public-private partnership

## Abstract

Insurance can, as has clearly been indicated in the literature, play an important role in dealing with catastrophe risks, not only as a compensation mechanism but also as a mechanism to influence the behaviour of the insured. It is the concept known as ‘insurance as governance’. However, we argue that there are limited possibilities for this role as far as the insurance of pandemics is concerned. The traditional technical tools, such as risk-based pricing, are difficult to apply. In addition, there may, ab initio, be serious problems in insuring pandemics within one of the main conditions of insurability (controlling moral hazard through an effective risk differentiation). One remedy that is traditionally applied, more particularly for natural catastrophes, is mandatory coverage. Furthermore, the capacity problem might potentially be solved through a multilayered approach in which, in addition to insurance and reinsurance, the government could also take up a role as reinsurer of last resort. That would also have the major advantage of stimulating market solution (and potentially providing incentives for the mitigation of damages), which clearly fails in a model where the government simply bails out operators. Finally, one important regulatory intervention is that insurers should be better informed than was apparently the case during the last pandemic about exactly which type of risks are covered and which are not.

## Introduction

Whether COVID-19 will turn out to be the most critical event in insurance history is still in question (The Geneva Association 2021a). The pandemic was unquestionably a catastrophe. It caused not only widespread sickness and death but also had financial consequences on a massive scale. According to the World Health Organization (WHO), the excess mortality associated with the COVID-19 pandemic was approximately 14.9 million in 2020 and 2021 (WHO 2022). In order to contain the COVID-19 crisis, lockdown measures have been widely adopted by countries across the world and the global economy has fallen into the deepest recession since World War II (The Geneva Association 2021b). For example, small businesses have lost USD 255–431 billion per month and more than 44 million workers were laid off due to government-ordered shutdown measures in the U.S. (French 2020). Research even indicates that pandemic risks will potentially cost as much as USD 23.5 trillion over the next 30 years (Hilsenrath 2020). The WHO declared the COVID-19 pandemic in March 2020 and found that the world was not ready for this type of low-probability, high-damage disaster (Swiss Re Institute 2022). Lloyd's predicted in 2008 that a pandemic was sure to occur at some point in the future (Lloyd’s 2008), and after the COVID-19 pandemic, it is widely accepted that the next pandemic is inevitable.

There is no doubt that pandemic risk prevention and loss compensation are at the centre of public and private agendas. One can imagine a variety of different mechanisms to compensate for the losses caused by a pandemic. One possibility is the use of liability under tort, although it may not be simple to find a liable tortfeasor and to prove causation. Attempts to use tort law for pandemic-related losses have so far not been very successful. A second possibility is to use social security and, more particularly (for employees), workers’ compensation. A large part of the losses caused by the pandemic (such as healthcare costs and losses due to inability to work) have been covered by social security, even though that is obviously only the case for jurisdictions that have a comprehensive social security mechanism in place. That may be more the case in the North (more particularly in Europe) than in the Global South. A third possibility (also often used in practice during the pandemic) is government compensation, mostly focusing on compensating businesses for losses due to business interruption and continuing (labour) costs. Finally, insurance could also play an important role.

As a leading risk management tool, insurance has been praised by legal and economics scholars and promoted for a much wider scale of events, more particularly, catastrophes (Hartwig et al. 2020; Hecht 2008; Kunreuther and Schupp 2021, “Insurance should be part of a risk management strategy to support businesses, non-profits, and local governments to address the risks they face – even if those risks are difficult to insure by traditional measures”). Insurance could potentially have at least two functions in relation to catastrophes in general, but also to pandemics. Firstly, it could protect against potential economic devastation by requiring individuals and businesses to insure against pandemic-related risks. This is the traditional function of *insurance as compensation*: based on the law of large numbers, it could compensate risk-averse individuals exposed to risky activities through risk pooling and risk shifting (see Kunreuther et al. 2013; Priest 1996). Besides compensation, insurance does more than just transfer risk, it also develops the function of *insurance as governance*: by providing incentives, insurers could impact policyholders’ behaviour and thus contribute to risk reduction (see, for example Ericson et al. 2003; Abraham 2013). Insurance could play a key role in regulating the conduct of policyholders by creating incentives for risk management and in some cases requiring or forbidding certain behaviour, and could even be a potent substitute for direct regulation by government. Increasingly, private insurance is seen as a tool to ‘outsource’ public governance (Ben-Shahar and Logue 2012).

When risk is considered catastrophic (such as terrorism or natural catastrophes) it is often considered uninsurable in private insurance markets. However, private insurance still plays an important role in many jurisdictions as it has become a substantial component of government programmes, mainly due to its governance function of managing risk, which includes risk assessment, mitigation and prevention (Klein and Weston 2020).^1^ The question therefore arises as to whether the solutions that have been worked out in the past for other catastrophes could be applied to the case of pandemics. Are pandemics inherently different than other disasters or can policymakers learn from the solutions worked out for other catastrophes? At first sight, so we will argue, the COVID-19 crisis might present a situation that does not comfortably fit either of the compensation or governance functions. Property insurers, especially those covering business interruption, maintained that private insurance coverage for economic losses caused by pandemics should be limited, otherwise they would go broke and the industry would be destroyed (Gründl et al. 2021; Hartwig et al. 2020). Insurers have consistently denied coverage, leading to substantial insurance litigation,^2^ as they apparently consider pandemic-related losses (more particularly losses due to business interruption) uninsurable. The central goal of our paper is to discover whether there is indeed a fundamental mismatch between insurance and pandemics, or whether under strict conditions and by applying tailor-made remedies, insurance might still play a role in dealing with pandemics.^3^

Our contribution is set up as follows: we first present the role of private insurance in insuring and governing COVID-19 pandemic risks in theory and then explain why it can, in practice, only play a limited role; next, we analyse the theoretical conditions of insurability and apply those to the COVID-19 pandemic in order to subsequently argue that in the case of pandemics it may be difficult to apply insurance as a governance mechanism. Finally, we suggest that there may be solutions to stimulate the insurability of pandemics and governance by insurance, provided an adequate legal framework is worked out. The final section concludes.

## The role of private insurance in insuring and governing COVID-19 pandemic risks

### Private insurance as governance: theory

Normally, governments play the central governing role, especially in times of crisis, because they have the authority and emergency powers to create and enforce laws and regulations (Vogel 2018; Gross and Ní Aoláin 2006).To contain COVID-19, many governments adopted shutdown measures, stay-at-home orders and mask mandates (Caulkins et al. 2022). However, governance, which “refers to all processes of governing, whether undertaken by a government, market, or network; whether over a family, tribe, corporation, or territory; and whether by laws, norms, power, or language” (Bevir 2013), is not limited to governments. It can be expanded to the whole of society, including markets, institutions and even individuals. Private governance systems are frequently discussed in socio-legal study, and are defined as “systems that promote long-term cooperation among individuals on the basis of social norms”.^4^ The use of private governance has generally been praised as socially efficient and optimal, provided that specific conditions are met.^5^

Private insurance as governance, whether considered as a subsection of private governance or a parallel field with similar underpinnings, implies that insurance is a contractual device that controls and motivates the behaviour of those insured in order to avoid the occurrence of losses (Ben-Shahar and Logue 2016). It is based on a contractual relationship and explores the potential value of insurance as a complement to, or substitute for, the state. There is increasing interest in the governance potential of insurance companies, both in the academic literature as well as at the policy level (Abraham and Schwarcz 2023, “[a] new vision for insurance – what we will call the ‘regulation thesis’ – is increasingly in vogue”).

The way in which this private governance by insurance companies functions is via the control of moral hazard (Shavell 1979). The insurer will assess the potential risk based on the past loss history (for example, through experience rating) in general, but also of that particular insured entity. For relatively new risks (when no reliable data and statistics are available) the insurer will use risk assessment to determine the premium and to ex ante adapt the premium as much as possible to the risk posed by the particular insured. When more information becomes available (obviously requiring a minimum loss frequency) the premium can be adapted to the individual situation of the insured (experience rating). All those ex ante and ex post techniques together should provide the insured with incentives to engage in risk-reducing measures.^6^ It is not only primary insurers that play a role as private regulators, reinsurance companies can also can act as ‘silent regulators’ (Abramovsky 2009), more particularly in exercising a governance influence on primary insurers (Mendoza 2014). The question obviously arises as to what extent it is possible for insurers to engage in this private governance, also in the case of a pandemic. This question will now be approached from various angles.

### A mismatch between COVID-19 pandemic losses and insurance in practice

It should be mentioned that insurance can potentially intervene to cover pandemic losses in many different ways. Some types of insurance coverage are more problematic in relation to a pandemic than others. Health and life insurance were less impacted by the pandemic than property insurers (Jerry II 2021). Insurance will undoubtedly be important as it covers medical expenses associated with injury and death under life and health insurance. However, those types of cover are general, i.e. irrespective of the cause of the disease or the resulting death. Insurers can in theory routinely incorporate pandemic risks into the price of their products with no material adverse impact on the availability of coverage (Hartwig et al. 2020). In other words, for those types of insurance, pandemics do not cause a significant problem (Hillier 2022, “There is no evidence of insurers withdrawing from the life insurance market, …, and excluding death due to COVID-19”), even though the losses may be higher than ex ante expected by the insurer.^7^ Most problematic from an insurance perspective is business interruption, as large losses arose from government lockdown orders (Knutsen and Stempel 2021; Muermann and Rothschild 2020).

The COVID-19 pandemic has caused widespread disputes about business interruption coverage. The question of whether an insurance programme is the best solution for problems caused by pandemics was even raised (U.S. House Committee on Financial Services 2020, “I question whether insurance is the best structure for this problem. Insurance is a system of risk transfer— it is not a system of economic assistance”). Insurers in many countries refused to pay pandemic-related claims due to tiny and optional pandemic coverage in business interruption insurance policies. For example, in France, the financial regulator stated that “93.3% of insurance policies did not cover the pandemic, 2.6% did and 4.1% were unclear” (del Carmen Boado-Penas et al. 2022). In China, the insurance regulator reported that as of June 2020, insurers had only paid an accumulated RMB 490 million in coverage for pandemic losses compared to the unprecedented impact on the economy (CBIRC 2021). In Germany, COVID-19 was not explicitly mentioned in the Infektionsschutzgesetz(2001)(IfSG) and provoked many court cases (del Carmen Boado-Penas et al. 2022). The extent to which a claim caused directly or indirectly by the virus is or is not covered has been the subject of debate.

For insurers, various grounds have been used in practice to refuse compensation for business interruption in many jurisdictions. Although the details of business interruption insurance policies vary, they generally turn on common legal issues of the interpretation and scope of coverage. For example, 164,178 out of a total 201,285 claims for business interruption losses caused by COVID-19 orders were closed without payment in the U.S. (NAIC 2020). According to the COVID Coverage Litigation Tracker, over 2000 lawsuits had been filed regarding business interruption insurance coverage in connection with the pandemic as of April 2022 in the U.S..^8^ A significant majority of federal cases were dismissed with prejudice, even in the state court, with more than 70% of decisions denying policyholders’ claims (Schwarcz 2022a).

To help disappointed policyholders, many governments proposed bills requiring retroactive insurance coverage for pandemics or mandating pandemic-related loss coverage. However, government efforts to solve the mismatches faced significant backlash and hurdles from the insurance industry.^9^ For example, the American Property Casualty Insurance Association (APCIA) opposed those proposals and argued that insurers are not in a position to cover these, and that retroactive payment for COVID-19 claims would bankrupt the industry (NAIC 2022a). As Sean Kevelighan, CEO of the Insurance Information Institute, stated: “[P]andemics are an extraordinary catastrophe… Pandemic-caused losses are excluded from standard business interruption policies because they impact all business, all of the same time” (Simpson 2020). It is as such understandable that the industry reacted against proposals to require retroactive insurance coverage, as they violate the basic principles of insurance. If ex post insurers were forced to cover losses that were ex ante not foreseen, they have not charged any premium for the particular risks, not set aside any reserves for future losses, nor required any specific measures from the insured aiming at the prevention of the losses. That is also why retroactive liability is generally considered uninsurable (Faure and Hartlief 2003).

Given the fact that some people will be hit more heavily than others in terms of health and their wealth, heightened awareness of pandemic-related risk and the potential function of insurance as governance and compensation all generate longstanding interest in insurance coverage against the losses resulting from pandemics (Nebolsina 2021, “the findings of the paper provide evidence that the demand for insurance services due to the COVID-19 outbreak in the United States can be expected to increase 2–6 times”). Although business interruption insurance against pandemics is not generally offered, there is still an increasing demand for welfare-improving insurance coverage in the case of a new pandemic (French 2020). After the COVID-19 pandemic, it might not be surprising that insurance systems fall short in supply of pandemic insurance. For example, an innovative parametric insurance policy launched in 2018 called PathogenRX providing specific coverage for pandemic-related losses for businesses is no longer available (Banham 2020). In addition, insurers will be much less enthusiastic about governing pandemic risks through rewriting a more precise pandemic/epidemic exclusion clause (compared to the currently disputed ‘virus exclusion clauses’).^10^

### A short summary

Theoretically speaking, insurance could play an essential role as governance in controlling moral hazard,^11^ even for catastrophic and systemic risks, such as natural disasters and financial crises (Heine and Faure 2012).^12^ When used for governance purposes, insurance can provide incentives to policyholders to prevent risk. In practice, concerning the COVID-19 pandemic, there seems to be a mismatch as insurers largely excluded pandemics from cover, either ex ante or ex post by rebuffing coverage claims. A ‘virus exclusion’, which is the regulatory tool that insurers use in business interruption insurance in relation to the COVID-19 pandemic, can be perceived as disreputable. This can have a similar effect to the ‘terror exclusion’ in property and casualty insurance just after the 9/11 terrorist attacks, since such governance tools become “an excuse for leaving expensive losses on powerless individuals” (Baker 2002). Refusal to cover and pay pandemic-related claims has eroded people’s trust in the insurance industry (del Carmen Boado-Penas et al. 2022). To some extent, so we will argue, this can be explained by the fact that it is challenging to fit pandemics within the traditional theoretical conditions of insurability.

## Insurability of pandemic risks

### Insurability in theory and its conditions

Insurance is, as we explained in the Introduction, potentially supposed to play a role in compensating pandemic losses and governing pandemic-related risks via risk management. From the perspective of the insured, insurance against pandemic losses will be purchased “if she considers the utility of a certain prospect of money income to be higher than the expected utility of an uncertain prospect of equal expected monetary value” (Cooter and Ulen 2008). From the previous section, however, it follows that there are apparently mismatches between insurance and pandemics. It is therefore natural to look for explanations for the mismatches and the absence of insurance as governance. Understanding the mechanisms of insurance that are, and are not, insurable when pandemics occur is critical to developing a promising approach to addressing the financial consequences of pandemics, since it affects insurers’ capability and willingness to provide pandemic-related products.

Many conditions or criteria for insurability or uninsurability have been discussed and explained in both the insurance actuarial and legal literature. For example, insurance economist Kunreuther (2008) proposed that if a risk is considered to be insurable, two conditions must be satisfied: the risk must be identified (condition 1), and a premium must be set for specific risks (condition 2). Holsboer (1995) looked for objective criteria of uninsurability, including availability of the product, insufficient coverage and lack of affordability. Some legal scholars insist that underwriting insurable risks involve three factors: rating risk properly, affordable premiums and measurable losses (see, for example Knutsen and Stempel 2021; Knutsen 2021).

Pandemics are undoubtedly considered a catastrophic or even a systemic risk. We distinguish four factors to consider whether pandemic-related catastrophe risks are insurable: (1) the predictability of risk, (2) the potential loss in the event that the risk materialises, (3) the random nature of risk and (4) the willingness of insurers. We will now review those four criteria and their applicability to pandemics and argue that, despite concerns and restrictions, under particular conditions, pandemic-related losses could, at least theoretically, be insurable.

*First*, the predictability of risk reflects the actuarial estimation. It requires that insurers can identify, quantify and estimate the frequency and severity of risks and the resulting losses (Berliner 1985; Swiss Re 2005). Insurance is in fact based on a very simple calculus: the actuarially fair premium that the insured should at least pay (of course increased with loading for administrative costs) should equal the probability (*p*) of the event multiplied by the potential damage (*D*) when the event occurs. Moreover, the totality of the premiums collected on that basis should in principle be sufficient (together with the reserves build up by the insurer) to compensate for the loss when the incident occurs. A crucial element is therefore that the insurer needs information (either on the basis of statistics or risk assessment) to calculate *p* and *D*. That may be a problem in the case of pandemic losses. Compared with traditional risks (such as auto liability risk), pandemic risks are regarded as emerging risks, which are associated with too much uncertainty to be predicted. In this case, it seems that pandemic risk is unmeasurable and unquantifiable due to the increasing frequency and severity of pandemics (e.g. the Spanish flu [1918–1920], the Asian Flu [1956–1958], the Hong Kong flu [1968] and the H1N1 flu [2009]) (Kunreuther and Schupp 2021). However, neither the size of the risk nor the potential loss estimates have prevented successful insurance operations in the past (Jaffe and Russell 1997). Looking back at insurance history, we can see plenty of examples of insurance against catastrophic losses that insurers could not predict in advance (Baker 2008). New risk statistics (based on past loss history) may be lacking, but that does not necessarily make the risk uninsurable as long as the insured can engage in risk assessment based on a modelling exercise. Moreover, insurers might add a risk premium to deal with ambiguity (see Hogarth and Kunreuther 1985; Kunreuther et al. 1993). With those techniques, catastrophic risks could also be insured in the past, even where data were lacking. One example is commercial satellite commercial aircraft insurance. These insurance products both involve huge losses, and at the initial stage of underwriting, insurers had no historical data to assess the intensity and the frequency of losses (Borch 1990; Jaffe and Russell 1997). What is more, with the development of big data technology, pandemic risk becomes easier to identify and quantify, and is thus moving away from a high-uncertainty risk profile (Mayer-Schoenberger and Cukier 2013).

*Second*, the potential loss of the risk refers to the insurer’s ability to pay the potential magnitude of catastrophe losses without running into danger of insolvency. In other words: the insurer needs capacity to deal with the loss once the pandemic emerges. Traditionally, insurers use a variety of techniques to increase capacity, such as co-insurance, reinsurance and securitisation (see Faure and Hartlief 2003), but these techniques also have their limits, especially in relation to catastrophes. The problem there is that many losses may occur at the same time, in which case an aggregation takes place that could confront an individual insurer with losses beyond its capacity (Faure and Bruggeman 2008). With respect to pandemic losses, insurers argued that if they were forced to provide COVID-19 coverage, the insurance industry would be destroyed (Knutsen and Stempel 2021). The most significant possible pandemic-related loss could threaten insurers’ solvency, and they would go bankrupt. As the magnitude of pandemic losses is significant, well above insurance-sector capital, pandemic risks violate most principles of insurability, and are therefore deemed ‘uninsurable’ by private markets (Insurance Council of Australia 2020; Schwarcz and Schwarcz 2014, describing the risk of global pandemics as a catastrophic risk). To address the catastrophe losses of pandemic risk, insurers could underwrite assessment insurance, which allows insurers to collect premiums after a loss if the insurance pool runs dry (Baker 2008). Furthermore, insurance companies could borrow outside capital through reinsurance or issuing insurance-linked securities to cover catastrophic claims (Schwarcz 2022b).

*Third*, the randomness of risk demands that risk events be accidental and uncertain in timing and location. The insurer can control the insured’s moral hazard and adverse selection for calculated risk (Khanna 2021). As a fortuitous event, the pandemic itself is unintended, unexpected and is not necessarily uninsurable (Knutsen and Stempel 2021). In May 2018, the largest reinsurer in the world, Munich Re, in cooperation with technology firm Metabiota, captured cutting-edge information on the pandemic and developed an innovative parametric insurance policy called PathogenRX, which provided specific coverage for losses caused by outbreaks, epidemics and pandemics.^13^

*Fourth*, there should be a market match between supply and demand. That means that insurers should have willingness (often referred to as ‘appetite’) to underwrite particular risks (in this case for pandemic losses), but that there should also be a willingness to pay on the side of the insured. The premium setting should moreover be such that insurers are able to make a profit with the particular product (Kunreuther 2008). If there is no willingness to pay on the side of the insured and therefore no substantial premium income to generate the capacity, the insurer might deny cover against pandemic losses. In particular, the already mentioned high correlation potential of the losses from pandemics might reduce insurers’ appetite to cover them (Kunreuther 2015). Moreover, there may equally be a mismatch between supply and demand, for example when insurers are ambiguous and risk averse and may therefore charge high (risk) premiums, whereas the insured assess the risk as much lower and therefore lack the willingness to pay the premiums offered by the industry. This is typically a case where supply and demand will not match and uninsurability emerges.

### Insurability in practice

In this section, we will further discuss the insurability of pandemic risks in practice. To be clear, insurability questions in the context of the COVID-19 pandemic have not so much arisen in health and life insurance, but rather in relation to property insurance.

Unlike business interruption, exposures of pandemic risk, for health insurance (of course, depending upon the size), do not necessarily pose a problem, especially in those states where most of the health costs are picked up by social security and the role of insurers is limited. As a result, the impact of the COVID-19 pandemic on private health insurance has been relatively modest. In addition, rising expenditure caused by the pandemic was offset mainly due to reductions in routine care costs (The Geneva Association 2020). For life insurance, in contrast with business interruption insurance, on the claims side, there are generally no exclusions for pandemics, although COVID-19 caused unanticipated excess mortality. On the business side, reduced longevity exposures in annuities essentially traded off increased life insurance compensation (Jerry II 2021). To summarise, pandemic risks have not posed fundamental health and life insurance insurability challenges.

We primarily focus on the insurability issue of business interruption insurance, which is the most problematic. Various forms of property–casualty insurance, especially business interruption insurance policies, limit coverage to losses resulting from general economic devastation. The APCIA, the primary national trade association for home, auto and business insurers, states that “[P]andemic outbreaks are uninsured because they are uninsurable” (APCIA 2020). It is property–casualty insurers that “have long maintained that insuring against large-scale pandemic risk is economically infeasible” (Hartwig et al. 2020). Insurers in court argue for the uninsurability of pandemics from the actuarial to the legal sphere, because “a nationwide lockdown had been an ‘inconceivable’ measure before 2020, and the actuarial equivalence principle does not work here.”^14^ Some insurance regulators share the same opinion as insurers. The Insurance Council of Australia conducted a study on insuring pandemics and concluded that a “traditional private sector insurance risk transfer solution to address pandemic risk [is] effectively impossible at this time” (Insurance Council of Australia 2020).

When reviewing these opinions of market players, one would tend to believe that pandemics are uninsurable for the simple reason that (as we also noted in the previous section) many insurers simply refuse cover, either ex ante or ex post. However, a de facto refusal to insure is not the same as uninsurability.

Insurability is not a binary concept (in the sense that the risk is either insurable or not), but rather a gradual, flexible concept explaining under which circumstances insurers would be more or less willing to provide cover for particular risks. If risks are uninsurable, insurers would rather not underwrite such policies. However, the boundary of insurability of specific risk is not set in stone. Like floods, terrorism and earthquakes, which are now fully or partially covered by insurance, pandemics are in the class of catastrophic risks (Gründl et al. 2021). As the American Academy of Actuaries (AAA) states, “pandemic risk is more similar to the catastrophic risks covered by programs like the Terrorism Risk Insurance Program and the National Flood Insurance Program than to risks normally insured by the commercial insurance market” (American Academy of Actuaries 2020).

### A short summary

One can notice that there is a kind of paradox: theoretically, pandemic losses may be difficult to insure, yet not necessarily uninsurable. In practice, however, insurers often consider pandemic losses as uninsurable and refuse cover. Unlike other catastrophes such as terrorist attacks, hurricanes and earthquakes, private insurers provide substantial coverage for the resulting losses (at least in developed countries), while coverage for pandemic losses, especially for business interruption, is extremely limited and has even become non-existent, due to the impact of COVID-19 (Hartwig et al. 2020). This casts shadows on pandemic insurance and raises concerns about insurability.

## The limits of insurance as governance

The starting point for our contribution is that insurance can play a role as a governance tool. However, some empirical studies show that in practice insurers do not always exercise that governance function in the way in which it is predicted in theory. This may more particularly be a problem in the case of pandemics as the possibility to deploy the technical tools necessary within the governance scheme may be limited.

### Empirical observations

As indicated above, we have reviewed the rise of private insurance as governance. Insurance can not only provide compensation after a covered accident or occurrence, but also be a form of *ex ante* private governance. When insurers underwrite risk, they should have incentives to mitigate that risk and any ensuing losses by influencing policyholders’ behaviour to reduce their payouts and thus help to reduce the risk and losses. Insurance could promote social welfare through the cumulative effect of reducing individual risks.

The facts do not always support the argument of private insurance governance. The notion and the effect of private governance are questioned by some lines of insurance practices, and show that insurance governance substituting for public governance is more limited than we expect.

Liability insurance is an up-and-coming field for insurance governance socio-legal research (Baker 2010). Extensive interviews with corporation directors and officers have bolstered arguments against the insurance as governance theory, “rather than discouraging bad behaviour by officers and directors of corporations, [directors and officers liability insurance] ‘ensures’ that corporate misconduct will occur” (Baker and Griffith 2010), dangerously undermining the deterrence function of shareholder litigation and the impact of securities laws. Professional liability insurance for civil rights claims against independent public school districts shows another type of failed governance (Mendoza 2020). Qualitative empirical evidence shows that “school district liability insurers attempt minor regulation through customary professional liability underwriting and loss prevention” (Mendoza 2020). The lack of private governance is due to insurers’ belief in market competition, the strength of districts’ local control desires and the inter-local risk pool’s political concerns about membership stability (Mendoza 2020). Food safety problems provide another vivid counterexample to the notion of insurance governance (Cogan Jr 2016).

In the field of catastrophe insurance, the governance role depends on the specific legal, political and economic context in which insurance schemes are embedded. Through in-depth empirical research, Krieger and Demeritt (2015) found that flood insurance schemes in Germany and the U.K. result in a pessimistic view of insurance in governing such catastrophe risks because of the disappointing performance in risk reduction and financial recovery (Krieger and Demeritt 2015). In some countries (more particularly Germany and Italy) there is often generous government compensation for victims of disasters, which may reduce incentives to purchase insurance. Epstein refers to this government compensation as “catastrophic responses to catastrophic risks” (Epstein 1996; see also Kaplow 1991). Gollier (2005) noticed that “solidarity kills the market”. Priest (1996) more particularly indicated that insurers could promote disaster risk reduction via the control of moral hazard. However, as a result of several regulatory failures, this governance via insurance often fails. Governance by the National Flood Insurance Program (NFIP) in the U.S. is subject to much more substantial moral hazard than the U.K. system, since it implicitly encourages people to live in flood hazard areas and undermines the private insurance market (Michel-Kerjan et al. 2015). Even in France, which has been praised for introducing comprehensive disaster insurance (Faure 2007), the expected incentivising effect of offering lower premiums to communities with a specific risk prevention plan is apparently lacking.

### Limited possibilities to employ technical tools in pandemic governance

Insurance as governance can only be played on the condition that an appropriate legal and regulatory framework assures well-functioning market operations. One of the most important conditions is the various potential technical methods to control the behaviour of the insured via adapted policy conditions (Shavell 1979). As a private regulator, insurance operates stealthily by using technical tools to reduce moral hazard (for example Heimer 2002). These technical tools, which almost all insurers use to one degree or another, include, but are not limited to, risk-based pricing, contract design (e.g. limits, deductibles, co-payments and exclusions), loss prevention, claims management and refusal to insure. However, pandemic insurance is incapable of promoting governance objectives because insurers face significant obstacles to deploying technical tools.

Risk-based pricing is considered the most basic technique for creating incentives to reduce risk (Ben-Shahar and Logue 2012). Insurers often adopt feature rating or experience rating to signal premium loss prevention. In practice, there are obstacles to applying risk-based pricing to control moral hazard. First, pandemic risks are still too complicated to understand and rating factors are based on correlation rather than causation; second, the availability of pandemic insurance is quite limited and insurers seldom communicate with the insured about rating factors; third, policyholders often do not have a meaningful capacity to adjust features, since insurance products for pandemics are usually too expensive for small businesses to purchase; fourth, experience rating is uncommon in low-frequency, high-impact coverage lines, such as pandemic risk, since the risks are uncommon and frequently influenced by factors beyond policyholders’ control; lastly, risk-based pricing may have a questionable impact where demand for pandemic insurance is limited due to affordability concerns and government bail out (NAIC 2022b). The fact that nobody bought the innovative pandemic insurance product PathogenRX launched in May 2018 also proves the above limits (Banham 2020).

The policy exclusion clause is regarded as an indirect way to regulate policyholders, as it excludes certain types of risk or claims from coverage. Compared to risk-based pricing, such a tool places a lower burden of information on insurers. However, when such an exclusion clause is not appreciated by the insured, its net effect is simply to shift the risk of loss onto the insured without reducing that risk (Schwarcz 2014). Take business interruption insurance, for example. Insurers deny pandemic-related claims mainly based on the wording of the insurance policy and insurance contract interpretation rules. Insurers refer to the presence of a virus exclusion clause that explicitly excludes viruses as a cause of loss and inhibits policyholders from pleading their pandemic-related compensation (French 2020). The insured oppose such an exclusion clause and argue that insurers only shift liability rather than reduce risks to society. The ‘myth of risk reduction’ or ‘moral hazard control wisdom’ through coverage restriction and exclusion clauses needs to be re-examined (Ben-Shahar and Logue 2012, but see Avraham and Porat 2021). The purpose of such actions by insurers is criticised by some scholars, “loss-reduction reduces accident frequency or magnitude––leading to a safer world—while loss-shifting only reduces the insurers’ liability under a policy for the accident without concern for accident frequency or magnitude. The difference is crucial, as loss-shifting does not decrease risk in the world and may instead create more risk” (Avraham and Porat 2021). Governance theory predicts that the shift from loss reduction to loss shifting will challenge private insurance because by combatting insureds’ moral hazard, insurers could endanger incentives to prevent accidents, which may have socially negative consequences.

The final technical tool used by insurers to regulate their insureds is the refusal to insure, which has similar consequences to but causes more controversy than the exclusion clause. Insureds in the U.S. complained that insurers’ refusal to insure became a refusal to pay from the very early days of the pandemic, since insurers started public relations campaigns and made use of media to distribute the message “there is no coverage for pandemic losses” (Knutsen and Stempel 2021). This seems to be a useful tactic for prospective litigants, and it impacts the legal sphere. However, such a public relation campaign might scare policyholders away since it amplifies insurers’ risk-leaning attitude to shift their own risk rather than reduce risks to society (Avraham and Porat 2021).

### A short summary

There are many reasons why it may be difficult to apply traditional technical tools. A pandemic is by definition a low-probability, high-impact event. This means that there is not a substantial loss history on which insurers can rely and therefore experience rating may be difficult. Moreover, given the low frequency of pandemics, there is also little possibility of learning. The traditional approach in insurance as governance is that via the control of moral hazard, insurers would provide incentives for self-protection ex ante. In the case of, for example, flooding, insurance would provide incentives, e.g. not to locate in flood-prone areas or to put expensive objects that are vulnerable to water in the attic rather than in the basement. In this way, insurance can play an important role in disaster risk reduction (see He and Faure 2022). However, it may be clear that in the case of a pandemic, the individual behaviour of an insured person may not affect the accident risk.

Insurance could still provide incentives to mitigate the damages; however, the possibilities for monitoring by the insurance company (by distinguishing good and bad risks) are extremely limited. It is, moreover, difficult to distinguish whether a business interruption (and the related losses) is solely the result of an exogenous factor (i.e. the pandemic) or whether endogenous elements (more particularly bad management) would have caused the losses as well. If that were the case, there is a danger that insurance leads de facto to the bailing out of poorly performing companies and may, moreover, even provide incentives for risky behaviour. The general problem is therefore that it may in practice be extremely difficult to cover only the losses due to pandemics, as business interruption is often a general cover, whereby the risk emerges that the insurer would also cover losses that would have happened anyway.

## Possible solutions within the legal framework

This paper should not be read as advocating the elimination of insurance in governance due to the discussion of its limits. Instead, it intends to complement the existing scholarship on private governance and, more importantly, stimulate discussion on how best to address the problems. However, the refusal of some insurance companies to pay pandemic-related claims, especially when it comes to business interruption insurance, has eroded trust in the insurance industry (del Carmen Boado-Penas et al. 2022).

### Regulating insurance policy wording

Private governance traditionally works based on social norms. Social norms are a great strength, but also the main weakness of private orders due to their open texture and rigidity (Griesel 2021). In contradistinction to norms, rule-based order is close-ended and flexible. Insurance can only fully play its role in governance on the condition that a legal framework is in place to facilitate it. A lack of legal rules means a lack of foundation for insurance markets since the capacity of private governance is bound by rules and ultimately grounded in law (Vogel 2018).

Business interruption has suffered most prominently from the pandemic’s impacts. Whether current business interruption insurance policies cover COVID-related losses is highly debated. The standard business interruption, or ‘time element’ insurance policy, taking the U.S. as an example, typically includes coverage clauses to the effect that “[W]e will pay for the actual loss of Business Income you sustain due to the necessary suspension of ‘your operations’… caused by direct physical loss of or damage to covered property…”, “caused by action of civil authority that prohibits access to the described premises”, “due to physical loss or damage at the premises of a ‘dependent property’ or a ‘secondary dependent property’” or “[I]f your ‘operations’ are suspended due to ‘contamination’.”^15^ In addition, as early as 2006, in response to the SARS pandemic, business interruption policies applied the ‘virus exclusion’ clause following the introduction of ISO, which often reads that “[W]e will not pay for loss or damage resulting from any virus, bacterium or other microorganism that induces or is capable of inducing physical distress, illness or disease.”^16^ Eighty-three percent of all business interruption insurance policies included an exclusion clause for viral contamination, virus, disease or pandemic (NAIC 2022a). The central issues stemming from the above policies’ terms are: (1) whether there is coverage for the businesses that suffered ‘direct physical loss or damage’; (2) whether there is coverage for the businesses that suffered from a government shutdown order under a civil authority provision; and (3) whether there is coverage for the business under the ‘virus exclusion’ clause (deLatour 2021).

Based on insurance policy interpretation rules in the U.S. (for a summary of the majority and minority rules of insurance policy interpretation rules, see American Law Institute 2018), insurers deny pandemic-related claims mainly based on the wording of the insurance policy and have mostly prevailed (Committee on Capital Markets Regulation 2021). First, insurers refer to the presence of a virus exclusion clause, which expressly excludes viruses as a cause of loss and inhibits policyholders from pleading their pandemic-related compensation (French 2020). Second, if the policy does not contain a virus exclusion clause, insurers further argue that COVID and government shutdown orders did not and could not cause any physical loss or damage to property, based on the clause that reads “the actual losses……caused by the direct physical loss of or damage to covered property” (Knutsen and Stempel 2021). Third, insurers refuse to compensate pure economic loss (for example income or profit losses) resulting from general economic downturn, which was encountered and denied for policyholders in lower Manhattan after the 9/11 terrorist attacks [Abraham 2011, “e.g. Duane Reade, Inc. v. St. Paul Fire & Marine Ins. Co., 279 F. Supp. 2d 235, 238-40 (S.D.N.Y. 2003): noting that the insurance policy in question required due consideration both to the experience of a drug store that had been destroyed by the 9/11 attack on the World Trade Center and to the probable experience thereafter had no loss occurred”].

In contrast, the U.K. invented a novel ‘test case scheme’, which could provide a better example than that of the U.S. to solve business interruption coverage disputes. The primary national regulator, the Financial Conduct Authority (FCA), formally filed a ‘test case’ on business interruption insurance with the court after the widespread outbreak of COVID-19. The selected policy wordings represented dozens of policy terms and were grouped under three different headings, containing the main types of clauses. The Supreme Court provided “authoritative guidance for the interpretation of similar policy wordings and claims” (FCA 2021) and largely affirmed the FCA’s motions.^17^ The FCA subsequently issued various directives to guide insurers’ claims payments, and virtually all policyholders with legitimate pandemic-related business interruption claims have been paid (Schwarcz 2022a).

To resolve future widespread coverage disputes, special rules in insurance law have been developed to reflect three fundamentals:

- Enhancing the governance role in reducing uncertainty and improving safety
- A consumer protectionist stance, because insurance companies hold the balance of power when determining the parameters of insurance coverage
- The importance of insurance as a compensatory safety net in society.

In the case of pandemic risks, these rules might (1) reconsider insurance contract interpretation rules to avoid insurers’ shifting rather than reducing loss, e.g. reasonable policyholder expectations of coverage for pandemic-related losses; ambiguity in property coverage for pandemic-related losses; causation, civil authority coverage and virus exclusion interpretation (Knutsen and Stempel 2021); and contractual manipulations and avoidance of the objection to risk-reducing technologies (Avraham and Porat 2021); (2) include rules on financial rewards provided by insurers for risk-mitigating measures of the insured; and (3) encourage insurers to supply information on risk management options to policyholders, thereby raising risk awareness as an important prerequisite for risk-mitigating actions.

### Mandatory rules on pandemic insurance coverage

Whether there is a legal or practical mandate for the purchase of insurance is important in the private governance of insurance. Many studies demonstrate that in workers’ compensation insurance, technical tools can reduce losses and promote safety (Ruser 1985; Barth et al. 2008). There is also proof that risk-based pricing and deductibles applied by auto insurers induce safer driving (Derrig and Tennyson 2011; Wang et al. 2008). In these successful stories, specific amounts of both workers’ compensation insurance and motor insurance are typically mandated by law. If the insured experiences significant rate increases due to past losses, they cannot simply drop or reduce coverage (Talesh 2012). Therefore, insurers operating in effectively mandatory lines of coverage, such as driver liability, homeowners and workers’ compensation, have strong reasons to see aggregate risk decrease, at least in the short and medium term (Abraham and Schwarcz 2023).

Mandatory coverage helps to manage adverse selection because it could prevent lower-risk groups from opting out of the pool. Mandatory insurance can also help to enhance damage mitigation. As Telesetsky (2010) argues, “the most important reason for mandating catastrophe risk insurance is to compel industry actors to take action under the supervision of the profit-motivated insurance industry”. With mandated private insurance, individuals who want to lower their insurance premiums would be likely to undertake mitigation measures.

Furthermore, behavioural experiments also show that individuals do not take insurance against low-probability, high-loss events such as pandemics, even if it increases their utility (Kunreuther et al. 2013). Behavioural problems like bounded rationality cause individuals to take an ‘it will not happen to me’ attitude and hence not purchase insurance coverage (Faure and Bruggeman 2008). Mandatory insurance is widely suggested as the solution to demand-side barriers (Bruggeman 2010). Also, for other types of disasters, comprehensive (mandatory) coverage has been advocated to deal with low demand due to cognitive and informational problems (Kunreuther 1968). Several countries, including France, Belgium, Norway and Spain, have now introduced such a mandatory compensation system for (particular) natural catastrophes (see further He and Faure 2018). Mandatory coverage therefore undoubtedly has the advantage of curing adverse selection by including all risks (good and bad) into the mandatory system; via premium and risk differentiation the moral hazard problem could be cured as well, “Only when government requires the purchase of insurance directly or indirectly, or when insurers adopt coverage terms akin to paradigmatic command-and-control regulation, does it make sense to label these techniques ‘regulation’” (Abraham and Schwarcz 2023).

However, mandating cover for pandemic losses also raises many questions. The first point is whether it would really be important to force all enterprises to take mandatory insurance for business interruption, whereas many (especially large) enterprises would certainly not have any demand for insurance. For large multinational enterprises of which the balance sheet is even larger than that of insurance companies, insurance for business interruption would only lead to an ex ante cost increase without added value. In theory, mandatory cover should also apply to those risks for which the added value of insurance would be obvious and where it could be argued that there is a market failure (i.e. small and medium-sized enterprises would not take insurance because of a lack of information). Moreover, mandating insurance for business interruption should obviously only be done when the moral hazard problem can adequately be solved. Given the fact that it may be difficult to distinguish business interruption caused by a pandemic from business interruption due to other causes (including mismanagement) it is questionable whether mandating insurance for business interruption generally would be a smart policy choice. Especially as this would potentially lead to high ex ante costs also for small and medium-size enterprises, it can be expected that this would equally lead to large political resistance (Depoorter 2006). As a result, it is not very likely that this will be effectively introduced.

### Public–private partnership (I): a multi-layered approach

Since mandatory coverage might be challenging for pandemics, a public–private partnership could be a viable solution. Similar to some existing programmes in the U.S., Japan and France, which provide coverage in the event of widespread disasters like floods, earthquakes and terrorist attacks, a multi-layered approach for pandemics may provide larger capacity and cover policyholders’ losses in the event of a future pandemic (deLatour 2021).

More importantly, to establish a feasible and sustainable public–private pandemic insurance partnership, the following two principles are relevant for utilising public–private partnerships as a risk-bearing tool that encourages policyholders to invest in cost-effective mitigation while at the same time solving issues of capacity and affordability. The first principle is to guarantee insurers as private risk regulators as we discussed above. The second principle is to take the government as an insurer of last resort and enable insurers to provide pandemic-related coverage. Such a multi-layered approach is also applied as far as natural catastrophes are concerned, where a first layer is often provided by insurers, a second by reinsurers and a third via reinsurance by the state (for examples see *inter alia* Bruggeman et al. 2012). We argue that it might be interesting to learn from this compensation model for natural catastrophes, and also as far as compensation for pandemic losses is concerned.

The multi-layered approach, we propose, could have sufficient capacity to be applied to the large potential losses caused by pandemics.

#### The first layer of losses is typically absorbed by the victims themselves

The first layer would provide mitigation incentives and prevent moral hazard since the victim bears a part of the costs.

#### The second layer of losses is covered by (mandatory) private insurance companies charging risk-based premiums

Under the multi-layered approach, the layers of risk transfer need to be supported by public- and private-sector activity centred on risk communication and reduction, especially on rating factors (Kousky and Kunreuther 2018). Meanwhile, assessment insurance, parametric insurance, reinsurance and securitisation could be invited and inform future approaches to catastrophe risk management for pandemics.

Even considering that normal risks (e.g. fire risk) or traditional catastrophe risks (e.g. terrorism and floods) are still smaller than those of pandemics, insurance history is full of what people in the insurance trade call *assessment insurance* (“With assessment insurance, the insurer has the ability to come back and collect more after a loss to help people who need it if the insurance fund runs dry”), see Baker (2008). Assessment insurance avoids insurers facing the same budget constraint that they used to.^18^ It is also a good argument against insurance companies’ legal perspective on insurability that the premiums “charged by insurance companies did not contain the possibility of protracted lockdowns, i.e. the customers did not pay for the risk of business interruption to the extent that was widely experienced during the COVID-19 pandemic” (see del Carmen Boado-Penas et al. 2022).

Parametric insurance (also called index-based insurance) is a ‘pre-valued’ policy and is widely proposed to address natural disasters. For example, the World Bank's Global Index Insurance Facility has been supporting the development of index-based disaster insurance for farmers and micro-entrepreneurs.^19^ Parametric pandemic insurance could provide a solution for business interruption losses and is not only good for the insured, but also significantly reduces the administrative claims burden of the insurer and allows for smaller payouts in return for payments policyholders can afford (Hillier 2022).

To solve the capacity concerns of catastrophe losses, insurers have traditionally protected themselves through private reinsurance contracts whereby portions of their losses from a catastrophic disaster are covered by some type of reinsurance arrangement (Kleindorfer and Kunreuther 1999). In addition, insurance-linked securitisation could be regarded as the process of transferring insurance risks from insurers and conveying them to third parties through tradable securities. Risk securitisation that utilises the ‘deep pockets’ of the global capital markets, and has a far greater capacity than insurance markets, could be used to help insure pandemic-related risks (Schwarcz 2022b). Securitisation is much like the parametric insurance approach rather than indemnity insurance, “[t]hrough the issuance of catastrophe bonds and through insurers covering a particular risk bonds can be sold that pay investors an attractive rate of return, unless loss exceeding an index or other proxy for a specified level of insured loss occurs” (Abraham and Baker 2022; NAIC 2022c).

#### The third layer of losses is covered by the public budget, whereby the government becomes a reinsurer of last resort

While at the second layer private insurance companies can only diversify risk horizontally between firms that are actually part of a risk pool, the government can diversify the third-layer risks over the entire population of firms and spread past losses to future taxpayers, creating cross-time diversification of risk that private insurance markets cannot achieve (Faure and Heine 2011). For example, the OECD estimates that by the end of 2022, government debt-to-GDP ratios in OECD countries will be approximately 20 percentage points higher than 2019 levels because of the COVID-19 crisis (The Geneva Association 2021a).

The government should promote and facilitate more robust insurance markets. Insurers do not currently offer pandemic insurance in practice due to the impact of COVID-19 (Schwarcz 2022a, b). Therefore, the pandemic insurance market could be regarded as the fabricating market, which may require deliberate market design by the government. The content of the private insurance market-enhancing framework has three pillars:

1. Sustaining a strong and capable government
2. Enhancing the market, while neither supplanting nor retarding it
3. Legalising the relationship between government and market to prevent the government from undermining well-functioning market operations (Paudel 2012).

For example, the government should help to solve market failures of the insurance market, secure insurers’ business operations using market mechanisms and promote insurance techniques as governance. What’s more, the government should help resolve future widespread coverage disputes. The U.K.’s Test Case Scheme and its success in facilitating the resolution of pandemic-related business interruption coverage disputes indicates that “public actors outside of the judiciary solely on identifying a set of pending coverage disputes …help to limit the uncertainty and costs produced by litigation like the pandemic BI coverage cases” (Schwarcz 2022a).

More importantly, the government should allow insurers to act as private regulators, thus reducing the risk and avoiding moral hazard and free riding, which (as the data show) have been spectacular in the case of government subsidies (Hudson et al. 2017). The reason is simple—the government provides undifferentiated lump sum payments, while insurers are able to differentiate risks in order to cure moral hazard.

Thus, the major advantage of this model would be that it puts the government in a different position: it would no longer just bail out enterprises, but rather stimulate insurers as private risk regulators, thus potentially also providing incentives for the mitigation of damages. The advantage of having this public–private partnership is that it could equally reduce direct government payments, which can create a huge moral hazard risk. Evidence from the payments made by governments for business interruption during the pandemic shows that there may have been a serious moral hazard risk. For example, in the Flemish Community in Belgium, 193,000 companies could count on government support of EUR 2.6 billion during the pandemic. In June 2022 an inspection was carried out showing that of the 23,234 files that were controlled, in 16,435 cases there was a wrongful payment. This led to a claim for reimbursement to the state of EUR 114 million.^20^

### Public–private partnership (II): timing and governance

To address the limits of private insurance governance, a new dynamic and structural public–private partnership should also be considered. The pandemic insurance programme would not function well without additional support from the state since it does not meet all the criteria of insurability, as we discussed above. Regulators have similar concerns that a “traditional private sector insurance risk transfer solution to address pandemic risk is effectively impossible at this time,” the “premiums would be high, and most likely unaffordable” and therefore, “government policy plays an important role in structuring solutions” (Insurance Council of Australia 2020). In addition, when the different roles of the government and the insurance market are clarified, it will be crucial and helpful to establish a well-functioning legal system for private insurance governance. The essential question for legislators is whether the pandemic insurance programme would be in the public interest and increase social welfare.

An important feature of such a new model is that the governance of disaster risks can be distinguished in three different phases whereby there are different roles for the government and insurers. Corresponding to a repetitive circle of catastrophe management as prevention–response–recovery (Cedervall Lauta 2015), we assess and allocate the ex ante, during and ex post compensation mechanisms (Dari-Mattiacci and Faure 2015; He and Faure 2021).

Ex ante is often regarded as the best way of disaster prevention and victim compensation, since it could reduce the damages caused by disasters and may even make compensation unnecessary. Insurance could work as the ex ante mechanism. Pandemic insurance transforms ex post liability and damages into ex ante costs (premiums), and could contribute to pandemic prevention and loss mitigation through, e.g. providing incentives for potential victims to escape from risk exposure (Ben-Shahar and Logue 2012).

*During* efforts are carried out when a state of emergency is declared. Government, rather than private (insurance) governance, plays a vital role *during* crisis management due to government efficacy capability in emergency management (He and Faure 2021). This type of government intervention has a legitimate justification due to its public good nature, especially in the immediate aftermath of pandemics. For example, on 23 January 2020, the Chinese government imposed a lockdown with other public health measures in Wuhan to quarantine an outbreak of COVID-19, which succeeded in suppressing a nationwide virus outbreak in China.^21^ Emergency relief is a popular practice as a *during* mechanism. In the U.S., pandemic relief bills, e.g. the Coronavirus Aid, Relief, and Economic Security (“CARES”) Act, were enacted and introduced government support programmes, e.g. the Small Business Administration’s Paycheck Protection Program, to address the economic impact of COVID-19 (Committee on Capital Markets Regulation 2021).^22^ The monthly basic income scheme was launched by the Spanish government, and the self-employment income support scheme was introduced by the British government for the most vulnerable households in 2020 (del Carmen Boado-Penas et al. 2022).

Ex post happens after the state of emergency is over, and its goal is mainly to compensate victims’ personal and property damages and return the conditions to those that would have existed had the disaster never occurred (Leonard and Howitt 2010). Government interventions including fund and recovery programmes, the liability rule of tort law, and insurance are all well-known ex post mechanisms for catastrophe victims. To effectively address economic devastation and subsequent business losses caused by pandemics, a multilayered approach is proposed in the above section.

## Concluding remarks

There has been an intense debate in the literature (also resulting in action by policymakers in various jurisdictions) concerning the way in which insurance could play a role in compensating victims of disasters. During the recent pandemic, the question about the extent to which insurance could equally play a role in compensating victims was also asked. Even though a pandemic can potentially cause a wide variety of losses, the most important ones that were the subject of a debate on insurability were business interruption losses. In many countries, most prominently in the U.S., at the very beginning of the pandemic insurers were quick to clearly state that, for a variety of reasons, business interruption losses would not be insurable under the applicable policies.

In this contribution we looked at the insurability of pandemics, not so much from a practical point of view (i.e. by examining what insurers have or have not de facto covered), but by taking a more theoretical approach concerning the insurability of pandemic risks. We started by noting that insurance can, as has clearly been indicated in the literature, play an important role, not only as a compensation mechanism, but also as a mechanism to steer the behaviour of the insured. It is the concept known as ‘insurance as governance’. However, we argued that there are limited possibilities for this role as far as the insurance of pandemics is concerned. Traditional technical tools, such as risk-based pricing, are difficult to apply given the fact that business interruption during a pandemic may have many causes, some of which may not necessarily be linked to the pandemic, but could, for example, have been caused by mismanagement. As a result, there may ab initio be serious problems in insuring pandemics, as one of the main conditions of insurability (controlling moral hazard through effective risk differentiation) may be very difficult to apply. Moreover, the magnitude of the losses caused by pandemics may be such that it outweighs the capacities of insurers.

However, notwithstanding the fact that it may be difficult to fit pandemics within the classic conditions of insurability, we explored whether some of the regulatory solutions applied to other types of catastrophes could provide a remedy for pandemics as well. We argued that this is the case only to a limited extent. One remedy that is traditionally applied, more particularly for natural catastrophes, is mandatory coverage. We argued, however, that this would make little sense for the case of business interruption losses as it would also force companies to purchase insurance for something for which the cover offers no added value. The capacity problem might be solved through a multi-layered approach in which, in addition to re/insurance, the government could also take on the role as reinsurer of last resort. That would also offer the major advantage of stimulating market solutions (and potentially providing incentives for the mitigation of damages), which clearly fail in a model where the government simply bails out operators. Finally, one important regulatory intervention is that insurers should be better informed than during the last pandemic about which types of risks are exactly covered and which are not. The problem apparently arose that many enterprises that had insurance against business interruption losses were assuming that their losses related to the pandemic would equally be insured, whereas, based on the wording of some policies, that was apparently not the case. Clear communication to policyholders with respect to the scope of coverage should avoid these types of mismatches. In sum, even though we remain cautious and argue that there are huge challenges in using the insurance as a governance mechanism in the case of pandemics (mostly because of potentially insurmountable moral hazard), provided that the scope of coverage can be narrowly defined and risks could be adequately differentiated, there can potentially still be a role for insurance.

## Data Availability

On behalf of all authors, the corresponding author states the data all cite the public sources, and can be made available.

## Footnotes

1 In the U.S., the Terrorism Risk Insurance Program and National Flood Insurance Program are both government-sponsored insurance programmes that include participation by private insurers.

2 See https://cclt.law.upenn.edu/ (last visited 20 May 2022).

3 To be clear, the role of third-party insurance plays a limited role in this context and, accordingly, we focus solely on first-party insurance. For example, according to the Association of British Insurers (2021), most COVID-19-related individual claims refer to first-party insurance and not to liability insurance claims.

4 Social norms are treated as laying the ground for private governance. See Griesel (2021).

5 “They promote economic efficiency by keeping governance lean and responsive to social needs. The law pales when compared with norms: it is blind to social realities, subservient to the rigidities of the legal system and incredibly costly.” See Griesel (2021). For examples of private governance of common pool resources, see Liu et al. (2018).

6 The possibilities of employing specific technical tools within this model of private governance in the case of a pandemic will be further discussed below.

7 When we refer to pandemics, we took the case of COVID-19 as an example. However, it may be dangerous to generalise this to any type of pandemic. From an insurance solvency perspective, some (future) pandemics could be more life threatening than COVID-19 and therefore endanger the viability of life insurers.

8 See https://cclt.law.upenn.edu/ (last visited 20 May 2022).

9 New Jersey was the first state to introduce legislation (A-3844), but ultimately it was put on hold. Others, e.g. The Gen. Assemb. of Pa., H.B. 2372, 2020 Sess. (Pa.2020) (“Business Interruption Insurance Act”); State of N.Y. Assemb., A. 10,226-B, 2020 Assemb. (N.Y. 2020) (“An Act in relation to requiring certain perils be covered under business interruption insurance during the coronavirus disease 2019 (COVID-19) pandemic”) faced significant backlash as well.

10 The prevalence of virus exclusions in policies is unclear, and legal decisions have been taken in favour of both insurers and policyholders. See Knutsen and Stempel (2021) and del Carmen Boado-Penas et al. (2022).

11 See *supra* Sect. "Private insurance as governance: theory". In addition, as far back as 1986, Reichman explored insurance as a social control tool to control crime risk. Ericson et al. (2003), adopted a sociological perspective to explore insurance as governance and documented how the insurance industry governs our lives and asserts insurance through nine interconnected dimensions.

12 For example, Ericson and Doyle (2004) applied their theoretical framework to four sets of risks (extending to catastrophic risks) governed by insurance: life, disability, earthquakes, and terrorism. We have conducted related research as well, e.g. He and Faure (2012, 2018).

13 https://www.marsh.com/us/campaigns/pathogenrx.html (last visited 20 May 2022). As we noted above, this excluded COVID-19 from coverage and is, moreover, no longer available.

14 For example, “[A] lawyer for insurance company QBEQBE.AX, stated during one of the hearings that the premia charged by insurance companies did not contain the possibility of protracted lockdowns, i.e. the customers did not pay for the risk of business interruption to the extent that was widely experienced during COVID-19 pandemic.” See del Carmen Boado-Penas et al. (2022).

15 See for example, Complaint Exhibit A at 68, JDS 1455, Inc. v. Society Insurance, No. 1:20-cv-02546 (N.D. Ill. Apr. 24, 2020). According to the survey, “[I]n some lines of insurance, all insurance companies provide identical coverage on the same take-it-or-leave-it basis.” See Randall (2007).

16 See Insurance service office, ISO FORM CP 01 40 07 06—Exclusion of Loss Due to Virus or Bacteria (July 6, 2006).

17 See generally Fin. Conduct Auth. v. Arch Ins. (U.K.) Ltd. [2021] UKSC 1 (appeal taken from Eng.).

18 Take Florida’s Citizens Property Insurance Corporation, for example. Citizens can secure emergency funding for catastrophic losses that exceed their own reserves under the assessment process. Citizens could impose a tax on all Citizens’ policyholders. Part of this assessment/tax is collected upfront, and part is spread out over a number of years, until the deficit is paid. See Ben-Shahar and Logue (2016).

19 Available at www.indexinsuranceforum.org.

20 https://www.vrt.be/vrtnws/nl/2022/06/09/114-miljoen-euro-onterecht-toegekende-coronasteun-teruggevorderd/, last consulted 14 July 2022.

21 https://en.wikipedia.org/wiki/COVID-19_lockdown_in_China (last visit 20 May 2022).

22 Pub. L. No. 116–136 (2020).

## References

[CR1] Abraham KS (2013). Four conceptions of insurance. University of Pennsylvania Law Review.

[CR2] Abraham, K.S., and Tom Baker. 2022. What history can tell us about the future of insurance and litigation after COVID-19. *DePaul Law Review* 71: 169–208.

[CR3] Abraham KS, Schwarcz D (2023). The limits of regulation by insurance. Indiana Law Journal.

[CR4] Abramovsky A (2009). Reinsurance: The silent regulator?. Connecticut Insurance Law Journal.

[CR5] American Academy of Actuaries (AAA). 2020. *Actuaries: Coverage of pandemic risk through property/casualty insurance could be designed like existing federal programs*. AAA. https://www.actuary.org/sites/default/files/2020-05/Actuaries-Coverage%20of%20Pandemic%20Risk%20Through%20Property%20Casualty%20Insurance%20Could%20Be%20Designed%20Like%20Federal%20Programs_0.pdf. Accessed 15 January 2023.

[CR6] American Law Institute. 2018. Chapter 1, Topic 1: Interpretation. In *Restatement of the law, liability insurance*. American Law Institute.

[CR7] American Property Casualty Insurance Association (APCIA). 2020. *Press Release, American Property and Casualty Insurance Association, APCIA releases new business interruption analysis*. APCIA. https://www.apci.org/media/news-releases/release/60052/. Accessed 15 January 2023.

[CR8] Association of British Insurers. 2021. *COVID-19: Insurers expect to pay up to £2.5 billion for UK insurance claims*. Association of British Insurers. https://www.abi.org.uk/news/news-articles/2021/02/covid-19-estimated-claims/. Accessed 15 January 2023.

[CR9] Avraham, R., and Ariel Porat. 2021. *Stacking the odds: How insurers make our world risker* (draft).

[CR10] Baker T (2002). Liability and insurance after September 11: Embracing risk meets the precautionary principle. The Geneva Papers on Risk and Insurance—Issues and Practice.

[CR11] Baker. T (2008). Embracing risk, sharing responsibility. Drake Law Review.

[CR12] Baker T (2010). Insurance in sociolegal research. Annual Review of Law and Social Science.

[CR13] Baker T, Griffith Sean J (2010). Ensuring corporate misconduct: How liability insurance undermines shareholder litigation.

[CR15] Banham, R. 2020. This insurance would have helped in coronavirus crisis but nobody bought it. *Insurance Journal*. www.insurancejournal.com/news/national/2020/04/03/563224.htm. Accessed 20 May 2022.

[CR16] Barth MM, Klein RW, Krohm G (2008). Workers’ compensation insurance experience rating and subsequent employer claims: The Wisconsin experience. Journal of Insurance Issues.

[CR17] Ben-Shahar O, Logue KD (2012). Outsourcing regulation how insurance reduces moral hazard. Michigan Law Review.

[CR18] Ben-Shahar O, Logue KD (2016). The perverse effects of subsidized weather insurance. Stanford Law Review.

[CR19] Berliner B (1985). Large risks and limits of insurability. Geneva Papers on Risk and Insurance.

[CR20] Bevir M (2013). A theory of governance.

[CR21] Borch K (1990). Economics of insurance.

[CR22] Bruggeman V (2010). Compensating catastrophe victims: A comparative law and economics approach. Journal of Environmental Law.

[CR23] Bruggeman V, Faure M, Heldt T (2012). Insurance against catastrophe: Government stimulation of insurance markets for catastrophic events. Duke Environmental Law and Policy Forum.

[CR24] Caulkins JP, Grass D, Feichtinger G, Hartl RF, Kort PM, Prskawetz A, Seidl A, Wrzaczek S, del Carmen Boado-Penas María (2022). COVID-19 and optimal lockdown strategies: The effect of new and more virulent strains. Pandemics: Insurance and social protection.

[CR25] Cedervall Lauta K (2015). Disaster law.

[CR26] China Banking and Insurance Regulatory Commission (CBIRC) (2021). Practices and experiences of China's banking and insurance industry in fighting the COVID-19 pandemic.

[CR27] Cogan JA (2016). The uneasy case for food safety liability insurance. Brooklyn Law Review.

[CR28] Committee on Capital Markets Regulation. 2021. *Pandemic business interruption insurance*. Committee on Capital Markets Regulation. https://home.treasury.gov/system/files/311/CCMR-Pandemic-BI-Report-July-2021.pdf. Accessed 15 January 2023.

[CR29] Cooter R, Ulen T (2008). Law and economics.

[CR30] Dari-Mattiacci G, Faure M (2015). The economics of disaster relief. Law and Policy.

[CR31] deLatour NE (2021). Insuring the “Uninsurable”: Business interruption insurance coverage and COVID-19. Georgia State University Law Review.

[CR32] del Carmen Boado-Penas M, Eisenberg J, Şahin Ş, del Carmen Boado-Penas María (2022). COVID-19: A trigger for innovations in insurance?. Pandemics: Insurance and social protection.

[CR33] Depoorter B (2006). Horizontal political externalities: The supply and demand of disaster management. Duke Law Journal.

[CR34] Derrig RA, Tennyson S. (2011). The impact of rate regulation on claims: Evidence from Massachusetts automobile insurance. Risk Management and Insurance Review.

[CR35] Epstein R (1996). Catastrophic responses to catastrophic risks. Journal of Risk and Uncertainty.

[CR36] Ericson RV, Doyle A (2004). Uncertain business: Risk, insurance and the limits of knowledge.

[CR37] Ericson RV, Doyle A, Barry D (2003). Insurance as governance.

[CR38] Faure M (2007). Financial compensation for victims of catastrophes: A law and economics perspective. Law and Policy.

[CR39] Faure M, Bruggeman V (2008). Catastrophic risks and first-party insurance. Connecticut Insurance Law Journal.

[CR40] Faure M, Hartlief T (2003). Insurance and expanding systemic risks.

[CR41] Faure M, Heine K (2011). Insurance against financial crises?. NYU Journal of Law and Business.

[CR42] Financial Conduct Authority. 2021. *Business interruption insurance*. FCA. https://www.fca.org.uk/firms/business-interruption-insurance. Accessed 15 January 2023.

[CR43] French CC (2020). COVID-19 business interruption insurance losses: The cases for and against coverage. Connecticut Insurance Law Journal.

[CR44] Gollier, C. 2005. Some aspects of the economics of catastrophe risk insurance. In *Catastrophic risk and insurance*, 13–30. Paris: OECD.

[CR45] Grisel F (2021). The limits of private governance, norms and rules in a Mediterranean fishery.

[CR46] Gross O, Ní Aoláin F (2006). Law in times of crisis: Emergency powers in theory and practice.

[CR47] Gründl H, Guxha D, Kartasheva A, Schmeiser H (2021). Insurability of pandemic risks. Journal of Risk and Insurance.

[CR48] Hartwig R, Niehaus G, Qiu J (2020). Insurance for economic losses caused by pandemics. The Geneva Risk and Insurance Review.

[CR49] He Q, Faure M (2018). Regulation by catastrophe insurance: A comparative study. Connecticut Insurance Law Journal.

[CR50] He Q, Faure M (2021). Compensation for victims of disasters: A comparative law and economic perspective. European Journal of Law Reform.

[CR51] He Q, Faure M (2022). Adaptation to climate change risks and regulation through insurance. Climate Law.

[CR52] Hecht SB (2008). Climate change and the transformation of risk: Insurance matters. UCLA Law Review.

[CR53] Heimer C, Baker T, Simon J (2002). Insuring more, ensuring less: The costs and benefits of private regulation through insurance. Embracing risk: The changing culture of insurance and responsibility.

[CR54] Heine K, Faure M (2012). Insurance against financial crises?. New York University Journal of Law and Business.

[CR55] Hillier R, del Carmen Boado-Penas M, Eisenberg J, Şahin Ş (2022). The legal challenges of insuring against a pandemic. Pandemics: Insurance and social protection.

[CR56] Hilsenrath, J. 2020. Global viral outbreaks like coronavirus, once rare, will become more common. *Wall Street Journal*.

[CR57] Hogarth R, Kunreuther H (1985). Ambiguity and insurers decisions. American Economic Review.

[CR58] Holsboer JH (1995). Insurability and uninsurability: An Introduction. Geneva Papers on Risk and Insurance.

[CR59] Hudson P, Botzen WJW, Czajkowski J, Kreibich H (2017). Moral hazard in natural disaster insurance markets: Empirical evidence from Germany and the United States. Land Economics.

[CR60] Insurance Council of Australia. 2020. *Insuring for pandemics study*. Insurance Council of Australia. https://insurancecouncil.com.au/resource/insurance-council-of-australias-insuring-for-pandemics-study/. Accessed 15 January 2023.

[CR61] Jaffe D, Russell T (1997). Catastrophe insurance, capital markets, and uninsured risks. Journal of Risk and Insurance.

[CR62] Jerry II, R.H. 2021. Insurance in a post-pandemic world: New and renewed challenges. *The Brief* 50 (4).

[CR63] Kaplow L (1991). Incentives and the government relief for risk. Journal of Risk and Uncertainty.

[CR64] Khanna A (2021). On insurability and transfer of pandemic business interruption risk.

[CR65] Klein RW, Weston H (2020). Government insurance for business interruption losses from pandemics: An evaluation of its feasibility and possible frameworks. Risk Management and Insurance Review.

[CR66] Kleindorfer PR, Kunreuther HC, Froot Kenneth A (1999). Challenges facing the insurance industry in managing catastrophic risks. The financing of catastrophic risk.

[CR67] Knutsen, E. 2021. The COVID-19 Pandemic and insurance coverage for business interruption in Canada. *Queen’s Law Journal* 46 (2): 431, 433–434.

[CR68] Knutsen ES, Stempel JW (2021). Infected judgment: Problematic rush to conventional wisdom and insurance coverage denial in a pandemic. Connecticut Insurance Law Journal.

[CR69] Kousky C, Kunreuther H (2018). Risk management roles of the public and private sector. Risk Management and Insurance Review.

[CR70] Krieger, K., and David Demeritt. 2015. *Limits of insurance as risk governance: Market failures and disaster politics in German and British private flood insurance.* Discussion Pater No: 80. Center for Analysis of Risk and Reduction, LSE.

[CR71] Kunreuther H (1968). The case for comprehensive disaster insurance. Journal of Law and Economics.

[CR72] Kunreuther, H. 2008. Insurability conditions. In *Encyclopedia of quantitative risk analysis and assessment*, 915–921. Hoboken: Wiley.

[CR73] Kunreuther H (2015). The role of insurance in reducing losses from extreme events: The need for public–private partnerships. Geneva Risk and Insurance Review.

[CR74] Kunreuther HC, Schupp J (2021). A framework for defining a role for insurance in “Uninsurable” Risks: Insights from COVID-19. Journal of Insurance Regulation.

[CR75] Kunreuther H, Hogarth R, Meszaros J (1993). Insurer ambiguity and market failure. Journal of Risk and Uncertainty.

[CR76] Kunreuther H, Pauly MV, McMorrow S (2013). Insurance and behavioral economics: Improving decisions in the most misunderstood industry.

[CR77] Leonard HB, Howitt AM, Kunreuther Howard, Useem Michael (2010). Acting in time against disasters: A comprehensive risk-management framework. Learning from catastrophes: Strategies for reaction and response.

[CR78] Liu J, Faure M, Mascini P (2018). Environmental governance of common-pool resources: A comparison of fishery and forestry.

[CR79] Lloyd’s. 2008. *Pandemic: Potential insurance impacts*. Lloyd’s. https://www.lloyds.com/news-and-insights/risk-reports/library/pandemic-potential-insurance-impacts. Accessed 15 January 2023.

[CR80] Mayer-Schoenberger V, Cukier K (2013). Big data: A revolution that will transform how we live, work, and think.

[CR81] Mendoza MA (2014). Reinsurance as governance: Governmental risk managements pools as a case study in the governance role played by reinsurance institutions. Connecticut Insurance Law Journal.

[CR82] Mendoza M (2020). The limits of insurance as governance: Professional liability coverage for civil rights claims against public school districts. Quinnipiac Law Review.

[CR83] Michel-Kerjan E, Czajkowski J, Kunreuther H (2015). Could flood insurance be privatised in the United States? A primer. The Geneva Papers on Risk and Insurance—Issues and Practice.

[CR84] Muermann A, Rothschild C (2020). “COVID-19: The economics of pandemic risks and insurance” of the Geneva Risk and Insurance Review. The Geneva Risk and Insurance Review.

[CR85] National Association of Insurance Commissioners (NAIC). 2020. *COVID-19 property and casualty insurance business interruption data call*. NAIC . https://content.naic.org/sites/default/files/inline-files/COVID-19%20BI%20Nat%27l%20Claims%20Aggregates_Oct_1.pdf. Accessed 15 January 2023.

[CR86] NAIC. 2022a. *Business interruption/business owner's policies (BOP).* NAIC (last updated 19 January, 2022a). https://content.naic.org/cipr-topics/business-interruptionbusinessowners-policies-bop. Accessed 15 January 2023.

[CR87] NAIC. 2022b. *Pandemics and COVID-19*. NAIC. https://content.naic.org/cipr-topics/pandemics-and-covid-19. Accessed 15 January 2023.

[CR88] NAIC. 2022c. *Parametric disaster insurance*. NAIC. https://content.naic.org/cipr_topics/topic_parametric_disaster_insurance.htm. Accessed 15 January 2023.

[CR89] Nebolsina E (2021). The impact of the COVID-19 pandemic on the business interruption insurance demand in the United States. Heliyon.

[CR90] Paudel Y (2012). A comparative study of public–private catastrophe insurance systems: Lessons from current practices. The Geneva Papers on Risk and Insurance—Issues and Practice.

[CR91] Priest GL (1996). The government, the market, and the problem of catastrophic loss. Journal Risk and Uncertainty.

[CR92] Randall S (2007). Freedom of contract in insurance. Connecticut Insurance Law Journal.

[CR93] Ruser JW (1985). Workers’ compensation insurance, experience-rating, and occupational injuries. RAND Journal of Economics.

[CR94] Schwarcz D (2014). Transparently opaque: Understanding the lack of transparency in insurance consumer protection. UCLA Law Review.

[CR95] Schwarcz D (2022). Redesigning widespread insurance coverage disputes: A case study of the British and American approaches to pandemic business interruption coverage. DePaul Law Review.

[CR96] Schwarcz S (2022). Insuring the ‘Uninsurable’: Catastrophe bonds, pandemics, and risk securitization. Washington University Law Review.

[CR97] Schwarcz D, Schwarcz Steven L (2014). Regulating systemic risk in insurance. University of Chicago Law Review.

[CR98] Shavell S (1979). On moral hazard and insurance. Quarterly Journal of Economics.

[CR99] Simpson, A.G. 2020. P/C insurers put a price tag on uncovered coronavirus business interruption losses. *Insurance Journal* (March 30, 2020). https://www.insurancejournal.com/news/nationa/2020/03/30/562738.htm. Accessed 15 January 2023.

[CR100] Swiss Re. 2005. *Innovating to insure the uninsurable*, 14. Swiss Re.

[CR101] Swiss Re Institute. 2022. *Endemic COVID: The end of the pandemic?* Swiss Re Institute. https://www.swissre.com/institute/research/topics-and-risk-dialogues/health-and-longevity/expertise-publication-COVID-19-the-end-of-the-pandemic.html. Accessed 15 January 2023.

[CR102] Talesh S (2012). Insurance law as public interest law. UC Irvine Law Review.

[CR103] Telesetsky A (2010). Insurance as a mitigation mechanism. Pace Environmental Law Review.

[CR104] The Geneva Association. 2020. An investigation into the insurability of pandemic risk. The Geneva Association Research Report. Author: Kai-Uwe Schanz. October. The Geneva Association. https://www.genevaassociation.org/sites/default/files/research-topics-document-type/pdf_public/insurability_report_web.pdf. Accessed 15 January 2023.

[CR105] The Geneva Association. 2021a. *The Global Risk Landscape after COVID-19: What role for insurance?* Author: Kai-Uwe Schanz. June. The Geneva Association. https://www.genevaassociation.org/research-topics/socio-economic-resilience/risk-landscape-after-covid-role-for-insurance. Accessed 15 January 2023.

[CR106] The Geneva Association. 2021b. *Public–private solutions to pandemic risk: Opportunities, challenges and trade-offs*. Author: Kai-Uwe Schanz. April. The Geneva Association. https://www.genevaassociation.org/research-topics/socio-economic-resilience/public-private-solutions-pandemic-risk-research-report. Accessed 15 January 2023.

[CR107] U.S. House Committee on Financial Services. 2020. *Insuring against a pandemic: Challenges and solutions for policyholders and insurers*, 33:56–34:07. U.S. House Committee on Financial Services.

[CR108] Vogel SK (2018). Marketcraft: How governments make markets work.

[CR109] Wang JL, Chung C-F, Tzeng LY (2008). An empirical analysis of the effects of increasing deductibles on moral hazard. Journal of Risk and Insurance.

[CR110] World Health Organization. 2022. *14.9 Million excess deaths associated with the COVID-19 pandemic in 2020 and 2021*. World Health Organization. https://www.who.int/news/item/05-05-2022-14.9-million-excess-deaths-were-associated-with-the-covid-19-pandemic-in-2020-and-2021. Accessed 15 January 2023.

